# Effects of User Fee Exemptions on the Provision and Use of Maternal Health Services: A Review of Literature

**Published:** 2013-12

**Authors:** Laurel E. Hatt, Marty Makinen, Supriya Madhavan, Claudia M. Conlon

**Affiliations:** ^1^International Health Division, Abt Associates Inc., Bethesda, MD, USA; ^2^Results for Development Institute, Washington, DC, USA; ^3^Johns Hopkins Bloomberg School of Public Health, Baltimore, MD, USA; ^4^Maternal and Child Health Division, United States Agency for International Development, Washington, DC, USA

**Keywords:** Access, Evaluation, Fees and charges, Healthcare quality, Low-income populations, Maternal health services

## Abstract

User fee removal has been put forward as an approach to increasing priority health service utilization, reducing impoverishment, and ultimately reducing maternal and neonatal mortality. However, user fees are a source of facility revenue in many low-income countries, often used for purchasing drugs and supplies and paying incentives to health workers. This paper reviews evidence on the effects of user fee exemptions on maternal health service utilization, service provision, and outcomes, including both supply-side and demand-side effects. We reviewed 19 peer-reviewed research articles addressing user fee exemptions and maternal health services or outcomes published since 1990. Studies were identified through a USAID-commissioned call for evidence, key word search, and screening process. Teams of reviewers assigned criteria-based quality scores to each paper and prepared structured narrative reviews. The grade of the evidence was found to be relatively weak, mainly from short-term, non-controlled studies. The introduction of user fee exemptions appears to have resulted in increased rates of facility-based deliveries and caesarean sections in some contexts. Impacts on maternal and neonatal mortality have not been conclusively demonstrated; exemptions for delivery care may contribute to modest reductions in institutional maternal mortality but the evidence is very weak. User fee exemptions were found to have negative, neutral, or inconclusive effects on availability of inputs, provider motivation, and quality of services. The extent to which user fee revenue lost by facilities is replaced can directly affect service provision and may have unintended consequences for provider motivation. Few studies have looked at the equity effects of fee removal, despite clear evidence that fees disproportionately burden the poor. This review highlights potential and documented benefits (increased use of maternity services) as well as risks (decreased provider motivation and quality) of user fee exemption policies for maternal health services. Governments should link user fee exemption policies with the replacement of lost revenue for facilities as well as broader health system improvements, including facility upgrades, ensured supply of needed inputs, and improved human resources for health. Removing user fees may increase uptake but will not reduce mortality proportionally if the quality of facility-based care is poor. More rigorous evaluations of both demand- and supply-side effects of mature fee exemption programmes are needed.

## INTRODUCTION

At the current average rate of change in maternal mortality ratios and neonatal mortality rates, only 31 developing countries will meet Millennium Development Goal 4 (MDG 4)−reducing child deaths−and still fewer (19 countries) will achieve MDG 5−reducing maternal deaths (1). With this slow rate of decline in mind, there have been calls for stakeholders across the globe to explore innovative approaches to achieving these goals, including the use of financial incentives aimed at both consumers and healthcare providers. User fee exemptions–targeted exemptions from out-of-pocket fees charged at some public-sector health facilities–are financial incentives that have both demand-side and supply-side effects; these influence the likelihood that consumers use health services as well as the volume and quality of services offered by providers. This paper reviews evidence on and the effectiveness of such fee policies in contributing to improved maternal and neonatal health.

User fees are often levied by governments to supplement their budget transfers to healthcare facilities in the context of under‐funded health systems. Most often, fee revenue is kept at the health facility level and covers local operating costs, including purchase of drugs, supplies, and salary supplements, although, in some contexts, governments may require that this revenue be transferred to the national treasury. Many sub-Saharan African countries instituted user fee policies in the early 1990s after the Bamako Initiative which called for community financing to augment resources available for primary care (especially drug supplies) and to improve quality and increase community involvement (2). Some early studies indicated that using user fee revenue to improve the quality of healthcare could increase demand for government health services (3).

The bulk of evidence has shown that user fees constitute an impediment to health service utilization among the poor (4-6), including facility-based delivery and emergency obstetric care. Fees also divert those who cannot pay to other (informal or private) sources of healthcare (7). To reduce the financial burden on patients and to increase access to healthcare services, many countries have again begun to reduce or eliminate user fees for certain services (fee exemptions), abolish fees for certain groups, such as pregnant women or under-five children (fee waivers), or abolish user fees entirely at primary healthcare facilities. These fee reductions have been put forward as an approach to increasing maternal health service utilization, reducing impoverishment, and ultimately reducing maternal and neonatal mortality.

While there have been several reviews of evidence on the effects of user fees and fee exemptions published in the past decades (8-10), including a recent Cochrane review by Lagarde and Palmer (11) and a special supplement to Health Policy and Planning focused entirely on user fee removal in November 2011, systematic reviews focusing on maternal and neonatal health specifically including both supply- and demand-side effects, have not been published. In 2012, the US Agency for International Development (USAID) commissioned a panel of economists and specialists in maternal and newborn health to conduct a literature review on the effects of user fee exemptions on maternal and newborn health outcomes, healthcare-seeking behaviours, and service provision, including both supply-side and demand-side effects. This paper summarizes the evidence review and addresses the following two focal questions:

- To what extent are user fee exemptions linked positively or negatively to maternal and neonatal health outcomes, the provision of maternal health services, or care-seeking behaviour by women?
- What contextual factors impact the effectiveness of user fee exemption programmes in improving maternal and neonatal health outcomes or the provision or use of maternal health services?

Standard economic theory suggests that, when prices are lowered, the quantity demanded will increase, and this relationship motivates user fee exemption policies. However, the interaction between price and the quality of services provided complicates an otherwise straightforward picture; in essence, the ‘product’ or health services on offer may change as the price drops, if there are fewer revenues available to motivate good performance of providers, purchase key inputs, like drugs, hire additional staff to handle increased volumes, etc. If both quality and the price drop simultaneously, the effects on quantity demanded are unknown. The extent to which suppliers of health services have the flexibility to accommodate increased demand (through hiring additional staff, for instance) can also, in turn, influence the quality of services. Finally, the presence of alternative providers offering affordable care at acceptable levels of quality will also influence consumer responses to price changes. The impact of user fee reductions on population health outcomes is mediated by how the demand for services and the quality of services respond to this policy change, making it essential to empirically measure these relationships in real-world settings.

## MATERIALS AND METHODS

We reviewed 19 papers addressing user fee exemptions and maternal health services or outcomes published since 1990. Eighteen original research articles were identified through the USAID-commissioned Call for Evidence, key word search, and screening process. Key words used included user fee*, user charge*, waiver*, exemption*, and out-of-pocket payment*, along with terms indicating the maternal and newborn population. The studies cover user fee exemption initiatives in Afghanistan, Burkina Faso, Ethiopia, Ghana, Mali, Nepal, Niger, Nigeria, Senegal, Sudan, South Africa, and Uganda. One study was dropped due to lack of pertinent information, and two additional relevant studies were identified and incorporated during the drafting of this manuscript. Teams of reviewers assigned criteria-based quality scores to each paper and prepared structured narrative reviews. The reviews identified the incentive provided, design of the evaluation of the incentive, quality of the evaluation, contextual factors influencing the incentive and the results attained, specific effects of the incentive on demand for and provision of maternal health services (including, where relevant, the quality of services), and any observed unintended consequences. Key findings and draft recommendations were vetted at the USAID-sponsored Evidence Summit in April 2012. A paper in this volume summarizes the Evidence Summit, with its methodology, article selection and review process in greater detail.

Three types of user fee policies were captured in this maternal health-focused review:

- User fees exempted or reduced for specific maternal health services only, such as antenatal care, facility-based delivery, caesarean section, or emergency obstetric care
- User fees waived for all services for pregnant women and newborns
- User fees abolished for all primary healthcare services, with effects measured among pregnant women and newborns.

The table summarizes the literature reviewed, including the country or region studied, the type and date of the user fee policy implementation, the geographic coverage of the policy and the study, whether user fee revenue was replaced or reimbursed (at least in theory), the evaluation methods used and date(s) of data collection, and whether the focus of the study was on demand- or supply-side measures.

## RESULTS

### Quality of evidence

The grade of the evidence was found to be weak in general, as was also noted in the recent Cochrane review (11). Few studies in this review had control or comparison groups; many of the studies reviewed were qualitative or cross-sectional in nature, and none of the studies used a randomized approach (Table). Few studies have rigorously evaluated the long-term effects of user fee exemption policies, although most such policies are fairly recent; most evaluations were conducted within one or two years after the policy change. In general, it is difficult to evaluate the relative merits of various approaches adopted by the countries in implementing user fee policies as the evidence is mixed and predominantly based on qualitative findings and weak designs.

The literature was especially limited for quantifying the effect of user fee exemption policies on supply-side indicators, such as the availability of drugs and supplies, workload and motivation of health workers, or quality of care. While the studies provided some descriptive information about supply-side effects, few documented objective quantitative measures.

### Effects of user fee policies on maternal health service utilization

The literature provides evidence that user fee removal for facility-based deliveries results in increased facility-based delivery rates but the evidence is weak. Penfold *et al*. (12) studied fee exemptions for delivery care at public and mission facilities in Ghana, which were introduced in 2003. They conducted a household survey in 2 regions after the policy had been in place for one (Volta region) or two years (Central region). Respondents reported a statistically significant increase (5 percentage points and 12 percentage points respectively) in recalled rates of delivering in a health facility, comparing the period before and after policy implementation. However, there was no comparison group.

De Allegri *et al*. (13) studied the effects of a government policy providing an 80% subsidy for facility-based deliveries in Burkina Faso. They conducted five repeated cross-sectional surveys of women in one rural district of Burkina Faso two years before and three years after the policy implementation. Over the five years, the proportion of facility-based deliveries increased from 49 to 84% of total deliveries. The authors attribute the change to the fee exemption policy; however, the trend was already increasing prior to the policy; there was no change in the slope of the trend; and there was no comparison group, making it difficult to draw robust conclusions. Senegal introduced user fee exemptions for normal deliveries and caesarean sections at health centres and hospitals in 5 poor regions in 2005. A facility survey conducted by Witter *et al*. (14) in six districts showed a statistically significant increase in facility-based deliveries from 40% (in 2004) to 44% (in 2005) of the expected deliveries in those districts. However, unbiased comparisons with non-implementing districts could not be made since the regions and districts were purposively selected.

**Table.** Summary of literature reviewed

Country (citation)	Type of fee policy (date of policy implementation)	Geographic coverage of policy/study	Fee revenue replaced?	Evaluation methods and study dates	Measured demand- or supply-effects?
Afghanistan (Steinhardt *et al.* 2011)	User fees abolished for basic package of primary healthcare services (April 2008)	National	No	Analysis of national health information system (HIS) data on deliveries and antenatal care (ANC) visits over 4 years (pre-post policy)% Review period: April 2005–June 2009	Demand and supply
Burkina Faso (De Allegri *et al.* 2011)	Exemptions for ANC (2002); partial exemptions for C-sections (2006), deliveries (2007)	National policy, study in 1 rural district	Yes	Post-only cross-sectional survey of women% Survey conducted in February–March 2009	Demand
Burkina Faso (Ridde *et al.* 2011)	Exemptions for ANC (2002); partial exemptions for C-sections (2006), deliveries (2007)	National policy	Yes	Qualitative policy analysis, analysis of HIS data from 8 districts over 5 years% Qualitative data collected in November 2008–April 2009; HIS data from January 2004–September 2009 analyzed	Supply and demand
Burkina Faso (De Allegri *et al.* 2012)	Exemptions for ANC (2002); partial exemptions for C-sections (2006), deliveries (2007)	National policy; study in 1 rural district	Yes	Analysis of 5 repeated cross-sectional household surveys on pre- and post-policy; no comparison group% Surveys conducted annually from 2006 to 2010	Demand
Burundi (Nimpagaritse and Bertone 2011)	Waiver of user fees for under-5 children and women giving birth (2006)	National	Yes, in theory	Descriptive case study% Observations of health facility director (2004-2008)	Supply
Ethiopia (Pearson *et al.* 2011)	Exemptions for ANC, delivery, postnatal care; hospital fee waivers for the poor (2005)	National	No	Post-only national health facility assessment% Assessment conducted in October 2008–January 2009	Supply
Ghana (Ansong-Tornui *et al.* 2007)	User fee exemptions for delivery care (implemented in January 2004 in Central region, April 2005 in Volta region)	Regional, then national; study in 2 regions	Yes, in theory	Pre-post audit of hospital-based delivery-related deaths, no comparison group% Review period: 24 months (Central) and 12 months (Volta) before and after policy	Supply
Ghana (Bosu *et al.* 2007)	User fee exemptions for delivery care (implemented in January 2004 in Central region, April 2005 in Volta region)	Regional, then national; study in 2 regions	Yes, in theory	Pre-post comparison of mortality rates for hospital-based deliveries, no comparison group% Compared 11 months (Central) and 12 months (Volta) before and after introduction of the policy	Supply
Ghana (Penfold *et al.* 2007)	User fee exemptions for delivery care (implemented in January 2004 in Central region, April 2005 in Volta region)	Regional, then national; study in 2 regions	Yes, in theory	Population survey of reported pre- and post- policy institutional delivery rates (conducted after policy); no comparison group% Survey conducted in April–May 2006; recall period 18 months (Central) and 6 months (Volta) before and after introduction of policy	Demand
Ghana (Witter *et al.* 2007)	User fee exemptions for delivery care (implemented in January 2004 in Central region, April 2005 in Volta region)	Regional, then national; study in 2 regions	Yes, in theory	Qualitative analysis of key informant interviews% Interviews conducted in September-December 2005	Supply
Ghana (Mills *et al.* 2008)	User fee exemptions for delivery care (implemented in 2004 in Upper East region)	Regional, then national; study in 1 district	Yes, in theory	Post-only cross-sectional survey of women who delivered in 2004% Survey conducted in October 2005–March 2006	Demand
Mali (El-Khoury *et al.* 2012)	Exemptions for C-sections (2005)	National	Yes	Analysis of national HIS data over 5 years (post-policy only), patients’ exit-interviews% Review period: 2005-2009; exit-interview data collected in 2010	Demand
Mali (Ponsar *et al.* 2011)	Exemptions for malaria care among pregnant women and under-five children in MSF* intervention areas (December 2006)	Pilot in 1 district	Yes	Pre-post analysis of programme monitoring data (intervention areas) and routine health centre data (non-intervention areas); review of financial reports% Review period: 2004-2008	Demand
Nepal (Witter *et al.* 2011)	Exemptions for delivery care (January 2009)	National	Yes	Pre-post analysis of facility records, no comparison group, purposive sample% Review period: 10 months before and after policy	Supply
Niger (Ridde and Diarra 2009)	Waivers for pregnant women and under-five children, funded by an NGO (2006)	Pilot in 2 districts	Yes	Case study/process evaluation% Data collected in April 2007	Supply
Nigeria's Kano state (Galadanci *et al.* 2010)	Exemptions for ANC, delivery, emergency obstetric care (2001)	Kano state	Partial	Analysis of hospital HIS data over 5 years (post-policy only), key informant interviews% Review period: 2001-2006	Demand and supply
Senegal (Witter *et al.* 2010)	Exemptions for delivery care and C-sections (January 2005 in 5 poor regions)	National, except Dakar; study in 5 regions	Yes, in theory	Mixed methods policy analysis: key informant interviews, focus group discussions, analysis of financial records, clinical record review% Review period: 2004-2006; data collected in November 2006–January 2007	Demand and supply
South Africa (Wilkinson *et al.* 2001)	Waivers for curative care for pregnant women and children below six years (1994)	National; study in 1 district	Not clear	Analysis of 1 mobile clinic's data over 6 years (pre-post policy), no comparison group% Review period: 1992-1998	Demand
Sudan (Abdu *et al.* 2004)	Exemptions for malaria care among pregnant women and under-five children (July 2001 to July 2002 only as part of experiment)	Pilot study in 8 facilities in 1 state	Yes	Pre-post experimental study with 6 experimental and 2 control facilities, pre-post household surveys; repeated patient-exit surveys during study, review of medical records% Baseline survey July 2001, endline July 2002; medical records from year prior to and year of study

*MSF=Médecins sans Frontières

Several studies relied on routine facility records or data from national health information system to document changes in facility delivery rates that might be associated with fee policies. A study in Nepal, which introduced free delivery care nationwide in 2009 (15), found a 19% increase in the number of institutional deliveries in 22 purposively-selected facilities, comparing the 10 months before and 10 months after initiation of the policy. In Nigeria's Kano state, which introduced free antenatal and maternity care at public secondary and tertiary hospitals in 2001, Galadanci *et al*. (16) report a 45% increase in the number of institutional deliveries over the subsequent 5-year period. No comparison group was tracked, however, and no pre-implementation data were presented.

There is some evidence that user fee exemptions for caesarean sections result in increased caesarean section rates. The study by Witter *et al*. in Senegal (14) reported an increase in caesarean section rates from 4.2 to 5.6% of facility-based deliveries over the one-year period after fees were removed. El-Khoury *et al*. (17) analyzed data of the national health information system from Mali, which removed user fees for caesarean sections in 2005. The national caesarean rate increased from 0.9% of estimated deliveries in 2005 to 2.3% in 2009; similar increases were apparent in each region of the country, although no pre-policy data are available. It is important to note that neither study is able to address whether the C-sections were medically necessary but the increases observed put C-section rates after fee removal well within the expected range for surgical deliveries of medical necessity.

User fee exemptions for malaria services led to increased utilization of facility-based malaria care by pregnant women in Sudan (18). In a quasi-experimental study of 8 randomly-selected health facilities in one state, fees for malaria care for pregnant women and under-five children were reduced by 0%, 25%, 50%, and 75% in 4 comparison groups. Exemptions from user fees were associated with increases in care-seeking for malaria at health centres, improved treatment-seeking behaviour, and earlier diagnosis for both children and pregnant women. Moreover, there appeared to be a dose/response effect with larger price reductions resulting in larger increases in use.

There is very limited and mixed evidence about whether removing fees for general curative care has positive or negative effects on the use of maternal health service. One study in South Africa (19) documented an unexpected decrease in the use of antenatal care service when fees for curative care were removed. The authors hypothesized that observed increases in congestion in clinics and the reduction in consultation times may have led to lower use of preventive care. However, a study in Afghanistan (20) found that removing user fees for other primary healthcare services (presumably increasing the workload of providers) did not appear to have any effect on facility-based deliveries or antenatal care visits.

As noted in the introduction, previous studies have documented that user fees disproportionately discourage the poor from seeking needed curative healthcare and that the poor use the coping methods that contribute to impoverishment to pay fees (6,21). The equity effects of removing user fees, however, are less clear as few studies have examined effects across wealth or income subgroups, especially with a maternal health lens. One recent study by El-Khoury *et al*. (17) analyzed patient-exit data collected from women who had received free caesareans in Mali in 2010 and analyzed reported asset ownership to estimate their socioeconomic status. The authors concluded that wealthier women were obtaining a substantially greater share of free caesarean sections than poor women, likely due to persistent geographical, cultural and transportation barriers to obtaining hospital-based care among the poor. However, no pre-policy data are available for comparison purposes. The study by Penfold *et al*. in Ghana (12) reported a non-significant finding that the removal of fees resulted in greater increases in facility-based delivery rates among the poor and less-educated women in Ghana, relative to other groups. Whatever the equity effects, several studies highlighted the fact that families still experience out-of-pocket spending even when maternal health user fees are nominally removed (14,22,23). This spending may be incurred for other costs within the facility (supplies, drugs), including fees for relevant and needed services that are not officially ‘covered’ by the exemption policy, i.e. informal fees or indirect costs for transportation and food.

### Effects of user fee policies on facilities, providers, and quality of care

An argument sometimes made in favour of user charges is that these could allow providers to improve the quality of care, using additional resources generated. This could, in turn, make providers more attentive to consumers since they are the source of the additional resources. Attentiveness to consumers and improvements in the availability of drugs and supplies could make the services sufficiently attractive that consumers would use as many services as those before the user charges were introduced. Akashi *et al*. in Cambodia (24) provided some indications that collecting user fees and putting the revenue towards supply-side improvements (purchases of drugs and supplies, hiring additional cleaners and security guards, and salary supplements) correlated with increased patient volumes for maternal health services.

On the other hand, if facilities experience an uncompensated loss in fee revenue while patient volumes simultaneously increase, the quality could decline over time. Shortages of inputs, like drugs and supplies could occur; providers may become less responsive and motivated; and consumers’ tendency to use more services at lower prices might be overcome by the perception of lower quality. The articles reviewed here provided mixed evidence of the effects of user fee exemptions on the quality of maternal healthcare provided: in 7 studies, quality was not measured; in others, the effects of exemptions were negative (5 studies), neutral/having no effect (5 studies), or mixed/inconclusive (2 studies). The most commonly-reported measures of quality were input-based (shortages of drugs and supplies). Other less-frequently reported measures included waiting times and time to receipt of care, use of partographs, post-operative infection rates, and case-fatality rates. Non-deleterious supply-side effects of user fee exemptions seem to correlate with whether policies were effectively put into place to ensure that facility-operating budgets and providers’ incomes did not decrease and whether systems-strengthening measures were implemented to accommodate increased patient volumes. The adequacy of pre-existing infrastructure, human resources, and supply chain systems was protective and so were the steps taken to reinforce systems prior to and during the implementation of the fee exemption policy (25).

A qualitative study by Witter *et al*. in Ghana (26) noted that the loss of user fee revenue at health facilities led to stock-outs of drugs and supplies, negatively affecting the quality of care provided and resulting in reinstituting fees by some facilities. Another article on fee exemptions, comparing a sample of hospital-based maternal deaths before and after delivery in Ghana, concluded that previously poor-quality delivery services remained as similarly poor quality after the introduction of the fee exemption policy (27). The Ethiopian National Emergency Obstetric and Neonatal Care Assessment of health facilities in 2008 found no difference in the quality of care between facilities that charged fees and those that did not (22), although there was a higher ratio of skilled birth attendants per delivery at facilities that charged fees, possibly because fee revenue was used for supporting better staffing.

Galadanci *et al*. (16) described increased workloads on health facility staff after user fees for deliveries were lifted in state hospitals in Kano state, Nigeria. There was no increase in remuneration to existing health workers and no increase in the number of health workers, resulting in reported decreased morale and performance of staff. While the authors did not provide quantitative indicators, they noted persistent problems with shortages of blood supplies, increased post-operative infections, and frequent stock-outs of drugs, such as oxytocin. Nimpagaritse and Bertone (28) described the sudden removal of user fees at health centres and hospitals in Burundi for all under-five children and women giving birth, from the perspective of the medical chief of a health district: the lack of preparation for the new policy resulted in critical negative consequences for healthcare providers, including stock-outs of drugs, reduced quality of services, disruption of the referral system, and reduced motivation of health workers.

In contrast, in the Médecins sans Frontières (MSF)-sponsored controlled intervention in Mali described by Ponsar *et al*. (29), MSF funds replaced the user fee revenues previously collected by health facilities for malaria care for pregnant women and under-five children. They directly supplied free rapid diagnostic tests and artemisinin-based combination therapy and paid a monthly sum to local clinic management organizations according to the number of staff, average operating costs, and measures of clinic performance. The authors conclude that quality was maintained and argue that specific attention to ensuring consistent drug supplies for remote areas is central to the success of user fee abolition measures. Ridde and Diarra (30) report on a user fee abolition initiative sponsored by a German non-governmental organization (NGO) in Niger, which similarly compensated health centres for the lost operating revenue and drugs as well as providing monthly bonuses to nursing staff. The supply-side effects here were mixed, however, with improvements in drug supplies but reports of increased negative providers’ behaviours towards patients. Concerns were raised about the sustainability of the (relatively large) bonuses after the NGO funding for them ends and the growth of a ‘parallel’ NGO-based fee exemption system.

The ways in which lost user fee revenue in facilities is replaced (or not) can directly affect providers’ behaviours and may have unintended consequences on motivation of providers. A case study by Witter and coauthors in 2011 on free delivery policy in Nepal (15) concluded that facilities appeared to have benefited financially from the fee reimbursements intended to replace user fees for delivery and noted that the reimbursements may be used for subsidizing other services. The authors posit (but do not have evidence) that incentive payments to health workers could lead to overprovision of some services in the future while fixed payments per case could lead to cutting corners in patient care. In Ethiopia where no government reimbursement is provided for lost fee revenue—and likewise at health posts in Senegal—many facilities simply continue to charge fees, despite official policies to the contrary (14,22). Witter *et al*. (14) also note that healthcare providers in Senegal are finding ways to pass on under-reimbursed costs to patients.

The studies reviewed here did not identify evidence that overprovision of caesarean sections was occurring in response to facility/provider reimbursements that replaced user fees (14,17) but this issue deserves close monitoring. Other studies have previously shown that fee-for-service reimbursement to providers can lead to unnecessary provision of caesarean sections (31,32).

### Effects of user fee policies on maternal and neonatal health outcomes

Only 3 studies in this review included any measurement of maternal or neonatal health outcomes (17,27,33). These studies were not designed nor powered to measure population-based changes in maternal or neonatal mortality, and the evidence is very weak. Bosu *et al*. (33) compared institutional maternal mortality (institutional maternal deaths as a proportion of institutional deliveries) during the year prior to and the year after the free delivery policy was introduced in two regions of Ghana. They reported 10% to 34% reductions in institutional maternal mortality in the two regions, although the decreases were not statistically significant. Statistics of the national health information system in Mali reported by El-Khoury *et al*. (17) indicated that the rates of post-caesarean maternal and neonatal death declined after the free caesarean policy was implemented. The study hypothesized that women sought emergency care sooner because of the policy but this could not be conclusively shown.

### Contextual factors

The second focal question for this review of evidence addresses contextual factors that could impact the effectiveness of user fee and exemption programmes. We interpreted ‘contextual factors’ to mean effect modifiers-factors external to the programme or policy which might interact with and alter intervention effects on maternal health service utilization and outcomes. The literature identified several such factors.

*Geography, distribution, and accessibility of infrastructure:* User fee policies will have limited effects on maternal health service utilization if services are not geographically accessible to populations in need (34). Fee exemptions do not overcome geographic barriers, weak transportation systems, or high transportation costs. Some countries have attempted to address transport barriers directly, such as through vouchers for transportation in Bangladesh (35) or establishing community ‘solidarity’ funds for emergency transport services as in Mali (17). The latter have had limited success, however, because of insufficient contributions from local municipalities and community members.

*Availability and expertise of health workforce:* As with other financial incentives aiming to increase service utilization, user fee exemptions will have little effect on maternal and neonatal health outcomes if the services that are free are not of adequate quality. Health gains “depend on having all of the components of skilled attendance available at the level of quality required to do more good than harm” (14). A sufficient quantity of health workers with appropriate midwifery and obstetrical skills is also critical, along with an appropriate distribution of those workers across both rural and urban areas.

*Availability, quality, and price of alternative (non-public) providers:* A change in the price of services at a public health facility could change where people decide to seek care, especially if there are many alternative providers available. Increased user fees at public facilities could spur more care-seeking from private-sector providers or informal/traditional care givers. Conversely, decreased user charges might attract users who otherwise would have used private providers. The effect of these choices on health outcomes depends on the quality of services provided by the alternative providers. If public-sector user fees decrease but the quality of services also suffers, especially if there are stock-outs of drugs and supplies, consumers may also choose to seek care from the private sector. Akashi *et al*. (24) noted that, while fees were increased in a public maternity hospital, prices were still less than that at nearby private facilities, thus preventing a decrease in the use of public facility.

*Governance and policy implementation capacity:* Successful user fee policy implementation at scale requires careful design, skilled management, and careful oversight. Some countries have experienced problems with the initial implementation and medium-term support for user fee exemption policies, specifically relating to replacing lost fee revenue to health facilities and ensuring clear communication about the policy. Ghana had substantial problems in disbursing funds to health facilities (26). Many facilities eventually stopped implementing the fee exemptions because of shortfalls in supplies and drugs. In Senegal, there were insufficient delivery kits available to facilities in the first year of the policy, and the distribution of kits did not mirror the needs of population (14). In general, reimbursing providers by giving them kits has been rife with challenges: kits are much less flexible than cash in meeting needs of the facilities; they require transport and stock management and do not cover labour costs. In Burkina Faso, Ridde *et al*. (36) concluded that the reduction of user fees was initiated before the groundwork was laid to make it a successful policy initiative. Confusion among health workers on the policy resulted in uneven implementation, and there were insufficient funds to subsidize activities across all health centres.

*Magnitude of non-service costs to consumers*: User fee policies only influence direct service costs, and posted fees for individual services may not constitute the largest share of costs faced by the family of a pregnant woman. The cost of associated supplies, medicines, food, and the indirect costs of other family caregivers attending the woman as well as transportation may exceed the magnitude of the consultation or procedural fee. In addition, where fee exemptions are implemented and poorly reimbursed, facilities may try to recoup lost fee revenue by raising other charges (23). Some facilities in Nepal, for instance, continued to charge families for items purportedly reimbursed by the Government (15).

*Relative prices of other services*: Removing fees for one individual service, such as caesarean section or delivery only, may have unintended consequences; this concern was recently echoed in a review by Richard *et al*. (23). Exempting fees for caesarean sections but not for normal deliveries, as in Mali, raises concerns about misaligned incentives to both users and providers (17). Removing fees for a package of maternal health services (and including transport vouchers to facilities) may be preferable, especially if the ultimate goal is to reduce maternal mortality and morbidity (37). If only fees for delivery care are removed (as in Ghana), there is a risk that mortality due to non-delivery pregnancy complications or postpartum issues would not change (26).

## DISCUSSION

Based on our assessment of this literature, the introduction of user fee exemptions appears to have resulted in increased rates of facility-based deliveries and C-sections in some contexts, although the evidence is weak and mainly from short-term, non-controlled studies. The introduction of user fee exemptions for malaria services resulted in increased rates of care-seeking for malaria among pregnant women, according to a quasi-experimental study, and greater fee reductions led to greater increases in care-seeking. Other than this analysis, we can say very little about the magnitude of potential effects on maternal health utilization due to user fee exemptions because of the weakness in study methods and the variety of policies and contexts reviewed. As yet, the impact of user fee exemptions on maternal and neonatal mortality has not been conclusively demonstrated; the introduction of user fee exemptions for deliveries may contribute to modest reductions in institutional maternal mortality but the evidence is very weak. Surprisingly, the effect of exemption policies on equity in the use of maternal health service has not been well-measured. It is not clear whether the poor benefit most from these policies, and it is known that merely subsidizing service costs for the poor is unlikely to eliminate inequity in healthcare utilization. Other barriers to service-use must be addressed to improve access for the poorest segment of women.

Although the effects of fee exemption policies on providers are not well-measured and the evidence is mixed, there is sufficient evidence to conclude that user fee exemption policies have important supply-side consequences, and these could negatively impact maternal health services if not carefully addressed. User fees contribute a non-trivial component of operating revenue of some health facilities; Nyonator and Kutzin (38) found that user fees in Ghana previously accounted for between two-thirds and four-fifths of the non-salary operating budget of government health facilities. Without replacement sources of revenue, shortages of drugs and supplies and reduced motivation of health workers may result in poor-quality care (negating health benefits of the policy) or facilities recouping costs from patients in alternative ways (negating the financial access benefits of the policy). In many of the examples reviewed here, fee revenue was theoretically supposed to be reimbursed or replaced by the government but, in practice, the replacement revenue was delayed, insufficient, or cumbersome to obtain [such as Senegal (14), Nigeria (16), Ghana (26), and Burundi (28)].

The evidence, thus, suggests that governments should link user fee exemption policies with the replacement of lost earnings and additional resources of facilities to respond to increased patient volumes after prices drop as well as with broader health system improvements, including facility upgrades, better transportation networks, and improved human resources for health. Few studies within the maternal health literature have documented how best to ensure that quality does not suffer after exemption policies are instituted; outside of the maternal health domain, studies, such as by Nabyonga-Orem *et al*. (39) in Uganda, have described interventions (such as additional budget transfers to districts, increased local flexibility in allocation of government funds, and institution of a pool system for commodities) that supported maintaining or even improving the technical quality of services. Identifying sustainable funding sources for fee replacement is critical. Several countries are using domestic funding for this purpose (Senegal and Burkina Faso); some countries largely relied on external funding (Nepal); some have relied upon a mixture of sources, including insurance (Ghana); and some have provided no replacement funding for lost fees, leaving facilities to continue collecting them (Ethiopia). It is unclear which of these financing approaches will be most sustainable over the long term.

For long-term sustainability, the literature (both within the maternal health domain and beyond) also indicates that user fee policy development and implementation must be done in a deliberate, carefully-planned manner. At a 2011 workshop in Bamako on maternal health user fee exemptions, sponsored by the Community of Practice on Financial Access to Health Services, participants from several West African countries also emphasized the importance of investing sufficient time in the policy formulation process for fee exemptions. This requires active participation of all stakeholders, including field-level practitioners and solid grounding in international and national scientific evidence (37). Hercot *et al*. (40) provide a useful framework for informing and evaluating the policy process surrounding implementation of fee exemption policy, noting crucial factors, such as careful planning of implementation steps, broad communication strategies targeted to different groups, commitment to the expected budgetary burden among government and international partners, and clear rules for transferring resources to health facilities to compensate for loss of income or new costs. Meessen *et al.* (25) reviewed policy processes for user fee removal in six sub-Saharan African countries (Burkina Faso, Burundi, Ghana, Liberia, Senegal, and Uganda) according to Hercot *et al*.'s framework. They highlight challenges, including insufficient preparation for the fee removal policy, poor design of the reform, and weaknesses in implementation processes. Relatedly, McPake *et al*. (41) illustrate proposed steps to forecast the impact of user fee removal on service utilization, estimate changes in needs of resources (human, material, and financial), mobilize those resources, and implement the policy reform.

### Limitations

The analysis was conducted through the lens of maternal health service utilization and outcomes; there is a larger body of evidence on user fee exemptions for other health services, which was, therefore, excluded as it did not address maternal and newborn health specifically. We referenced some of that literature here but cannot claim to have reviewed the full literature rigorously.

### Conclusions

This review has highlighted potential and documented benefits (increased use of maternity services) as well as risks (decreased provider motivation and quality) of user fee exemption policies for maternal health services. Our policy recommendations (Box 1) are limited, given the general weakness of the evidence. A clear message is that removing user fees may increase uptake but will not reduce mortality proportionally if the quality of facility-based care is poor. Additional research on approaches for reducing demand-side barriers without hurting the quality of maternal healthcare is needed; the recent review by Richard *et al*. emphasizes these research needs as well (23). In general, more robust evaluations of user fee policies are needed with adequate sample-sizes, appropriate comparison groups, stronger quantitative measurement of supply-side impacts, robust quality indicators, and continued use of qualitative methods to document policy implementation processes (Box 2). This will improve the quality of information on which to build cost-effective interventions, interventions that will reach and provide lifesaving care for millions of women and newborns while accelerating progress towards Millennium Development Goal 4 and 5.

**Box 1. Policy recommendations**

- Because user fees disproportionately limit access to priority maternal health services and cause financial burden among the poor, policies should be put into place to limit these effects. Fee exemption may be a short-term approach in contexts where broader risk pooling or prepayment schemes are not yet in place. These may increase uptake of facility-based delivery care, care for during pregnancy, and C-sections. However, exemption and waiver programmes should be designed and implemented carefully, with attention to avoiding potentially detrimental supply-side effects that could negatively impact the quality of maternal healthcare and limit the beneficial effects of increased access.
- Governments that wish to eliminate or reduce fees at the point of service should carefully design a system for replacing lost user fee revenue to facilities and providers, to avoid unintended consequences (including overcrowding, decreased quality of service provision, and the charging of informal fees). They should invest sufficient time in the policy formulation process, involving key practitioners from the field as well as those stakeholders that can identify and mobilize long-term sources of funding.
- To maximize value for money, policy-makers should aim to target financial incentives, like user fee exemptions to the poorest groups since they are most affected by price barriers. Conserved resources could be allocated to compensate providers and support quality improvements.
- Policy-makers should link policies that incentivize the use of maternal health services with broader improvements in the health system, including facility upgrades, ensured supply of needed diagnostics and drugs, better transportation networks, transportation subsidies, and a sufficient number of trained, deployed, equipped and motivated health workers.
- Fee exemption policies should be implemented as part of a broader, coordinated framework for health financing that aims ultimately towards risk-pooling and universal health coverage. Since reimbursing providers, ensuring quality, and promoting financial protection entail system-wide reforms, fee policies should be part of an overall health financing strategy rather than stand-alone interventions.

**Box 2. Research recommendations**

- *Stronger study designs for user fee policy evaluations:* Researchers should strive to ensure plausible comparison groups in observational designs and use experimental or quasi-experimental designs (e.g. randomizing health facilities or districts) wherever possible.
- *Longer time horizons:* There is a need for evaluations of more mature user fee exemption policies to identify longer-term effects on maternal health utilization, outcomes, and service quality. Early evaluations give an incomplete picture, especially as both provider and consumer behaviours may adapt to the policy over time, and initial effects may dampen or may not persist.
- *Implementation research and documentation:* During implementation of fee exemption policies, implementers should prioritize process documentation and increase the use of qualitative methods to answer the ‘how?’ and ‘why?’ questions. Operations research is particularly needed to determine how health workers should be incentivized to provide good-quality care in the absence of user fee revenue.
- *Equity and targeting:* There is a need to measure the impact of user fee exemptions on equity of access to maternal healthcare and of distribution of healthcare resources across socioeconomic groups, between rural and urban women, and for marginalized groups. The question of how best to target exemptions to priority subgroups also needs continued study.
- *Cost-effectiveness of different exemption approaches:* Key cost-effectiveness questions are unanswered for maternal health services. Given the limited resources, is it most cost-effective to exempt individual high-impact or high-cost services (such as caesarean sections), a package of services (antenatal, delivery, and postnatal care), a component of care (medicines), a targeted group (low-income pregnant women, high-parity women, rural women), or an entire population group (all pregnant women)?
- *Cost-effectiveness relative to other demand-side approaches:* Given the limited resources, it is critical to understand the cost-effectiveness of user fee exemptions in relation to other options that are demonstrated to increase the use of maternal health services (such as vouchers, conditional cash transfers, or insurance). Little evidence addressing this question is available.

## ACKNOWLEDGEMENTS

This paper was prepared, in part, based on initial reviews conducted by a panel of experts for the US Government Evidence Summit on Enhancing Provision and Use of Maternal Health Services through Financial Incentives. In addition to the authors, these readers included Isabella Danel of the Centers for Disease Control and Prevention, Karen Fogg of USAID, Ana Langer of the Harvard School of Public Health, Craig Lissner of the World Health Organization, and Ubaidur Rob of the Population Council. Sharon Nakhimovsky of Abt Associates contributed to an earlier version of this manuscript. We gratefully acknowledge the guidance and critical feedback provided by Marjorie Koblinsky at USAID. Preparation of this manuscript was funded by USAID through the Health Finance and Governance Project.
